# Polymer Optical Waveguide Grating-Based Biosensor to Detect Effective Drug Concentrations of Ginkgolide A for Inhibition of PMVEC Apoptosis

**DOI:** 10.3390/bios11080264

**Published:** 2021-08-06

**Authors:** Chunxue Wang, Pengfei Yi, Jiang Li, Haibing Dong, Changming Chen, Daming Zhang, Haiqing Shen, Bendong Fu

**Affiliations:** 1 College of Veterinary Medicine, Jilin University, Changchun 130062, China; wcx20@mails.jlu.edu.cn (C.W.); lijiang20@mails.jlu.edu.cn (J.L.); chxndhb@163.com (H.D.); shenhq@jlu.edu.cn (H.S.); fubd@jlu.edu.cn (B.F.); 2 State Key Laboratory of Integrated Optoelectronics, College of Electronic Science and Engineering, Jilin University, Changchun 130012, China; chencm@jlu.edu.cn (C.C.); zhangdm@jlu.edu.cn (D.Z.)

**Keywords:** waveguide grating-based biosensor, polymer optical waveguide, effective drug concentrations

## Abstract

In this work, we successfully developed a fluorinated cross-linked polymer Bragg waveguide grating-based optical biosensor to detect effective drug concentrations of ginkgolide A for the inhibition of pulmonary microvascular endothelial cell (PMVEC) apoptosis. Fluorinated photosensitive polymer SU-8 (FSU-8) as the sensing core layer and polymethyl methacrylate (PMMA) as the sensing window cladding were synthesized. The effective drug concentration range (5–10 µg/mL) of ginkgolide A for inhibition of PMVEC apoptosis was analyzed and obtained by pharmacological studies. The structure of the device was optimized to be designed and fabricated by direct UV writing technology. The properties of the biosensor were simulated with various refractive indices of different drug concentrations. The actual sensitivity of the biosensor was measured as 1606.2 nm/RIU. The resolution and detection limit were characterized as 0.05 nm and 3 × 10^−5^ RIU, respectively. The technique is suitable for safe and accurate detection of effective organic drug dosages of Chinese herbal ingredients.

## 1. Introduction

Ginkgolide A, a form of traditional Chinese medicine (TCM), can be used as a potent and specific antagonist to platelet-activating factor (PAF) [1]. As an active organic drug biosynthesized in the plant, ginkgolide A can prevent thrombus formation, bronchoconstriction, and allergic reactions [2]. In particular, it has been shown that ginkgolide A can significantly reduce pulmonary histological damage and effectively inhibit pulmonary microvascular endothelial cell (PMVEC) apoptosis, which is advantageous to reduce acute lung injury (ALI) [3]. In this case, it is important to precisely select effective drug concentrations of ginkgolide A in order to realize the scientific biomedical application of TCM.

The liquid chromatography technique is a main detection method for analyzing drug monomers, but this technology also has some limitations, such as the long sample preparation time, multi-step operation, and high cost [4,5]. In comparison, optical waveguide sensors with high sensitivity, high integration, and high compatibility have been proposed to achieve highly precise biomedical detection, aimed at special selection of matter based on lab-on-chip application [6,7,8,9,10]. Some typical waveguide structures have been used to define sensors such as interferometers [11], resonators [12], and gratings [13]. In contrast to other waveguide structures, grating-based sensors are more suitable for high sensitivity and fast detection of label-free solution or gas [14,15,16]. In particular, polymer waveguide grating-based sensors with better manual control, more fabricating flexibility, and more targeted selection have been used to realize state-of-the-art integrated functional sensing chips [17,18]. Furthermore, polymer waveguide materials with specific functional groups are more advantageous for selecting organic adhesion monomers and capturing small drug molecules from TCM solutions by multi-hydrogen-bonding interacting effects [19,20].

In this work, a fluorinated copolymer waveguide grating-based optical biosensor to detect effective drug concentrations of ginkgolide A for the inhibition of PMVEC apoptosis was designed and fabricated. Low-loss fluorinated SU-8 (FSU-8), as the sensing core layer, and polymethyl methacrylate (PMMA), as the sensing window cladding, were synthesized. Adhesion and capture characteristics between ginkgolide A and polymer waveguide materials were analyzed by the molecular docking technique. The effective drug concentration range of ginkgolide A for inhibiting PMVEC apoptosis was studied. Sensing structures and properties of the Bragg waveguide grating-based biosensor were designed and simulated. The fabrication process of the device is given, the actual structural size of the waveguide gratings is characterized, the sensitivity of the biosensor is measured, and the resolution and detection limit of the chip are obtained. The technique will be useful to ensure the effectiveness and safety of organic drug dosages efficiently and accurately.

## 2. Experiment

### 2.1. Sensing Polymer Waveguide Materials

**Figure 1:**
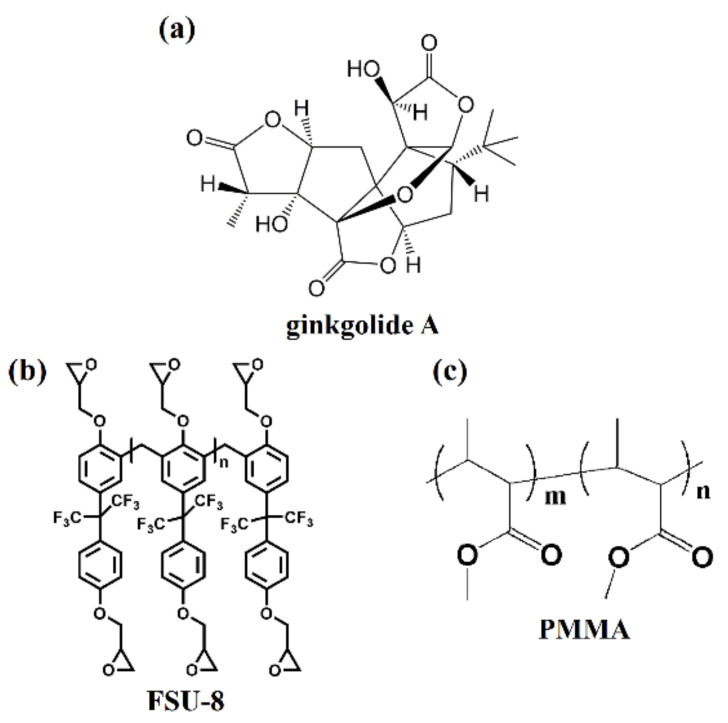
Molecular structure of (**a**) ginkgolide A, (**b**) FSU-8, and (**c**) PMMA.

Ginkgolide A was obtained from Longhua Technology Co., Ltd. (Luoyang, China). The molecular structure of ginkgolide A is shown in Figure 1a. FSU-8 and PMMA were self-synthesized and used as waveguide core and cladding materials, respectively, for the sensor. The molecular structures of FSU-8 and PMMA are shown in Figure 1b,c. Parts of the C–H bonds in the FSU-8 were displaced by C–F bonds, which could effectively reduce the optical absorption loss of the polymer in near-infrared wavelength bands [21,22]. The technique is advantageous for realizing relatively low transmission loss in the 1550 nm signal wavelength for the waveguide biosensor.

**Figure 2:**
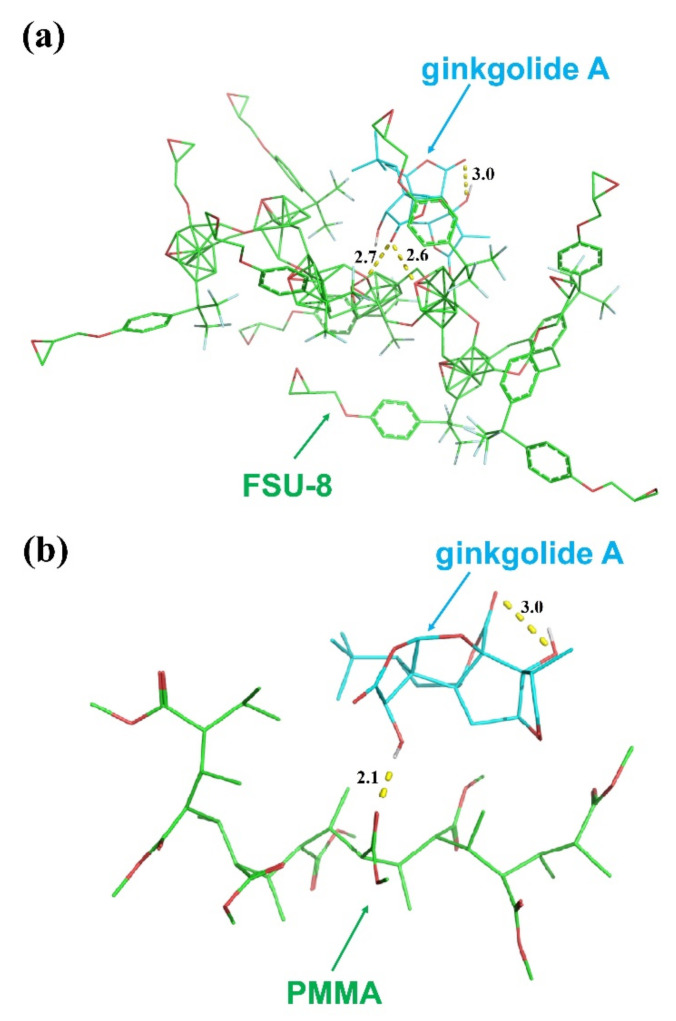
Molecular docking model simulation of (**a**) ginkgolide A and FSU-8 and (**b**) ginkgolide A and PMMA.

Adhesion and capture characteristics between ginkgolide A and the polymer waveguide materials were analyzed by molecular docking. Molecular docking models based on FSU-8 and PMMA with ginkgolide A were simulated by AutoDock software, as shown in Figure 2. FSU-8 and PMMA could capture the organic monomer of ginkgolide A effectively by hydrogen-bonding affinity. It was found that the binding energy between FSU-8 and ginkgolide was −6.35 kcal/mol (−26.57 KJ/mol) and between PMMA and ginkgolide A was −3.47 kcal/mol (−14.52 KJ/mol). It is obvious that hydrogen bonding forces and binding energy are stable and strong between the polymer waveguide materials and ginkgolide A, which is useful for realizing high detection sensitivity of the optical biosensor.

### 2.2. Biosensor Design and Fabrication

**Figure 3:**
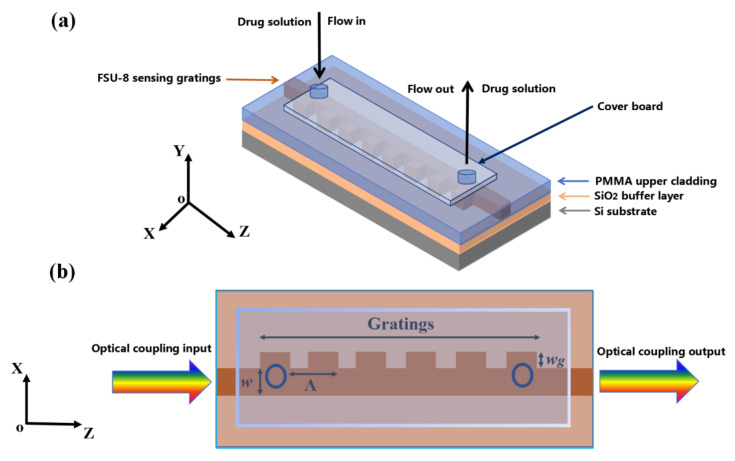
Structural schematic diagram of grating-based biosensor: (**a**) 3-D framework and (**b**) X-Y plane.

To realize the optical biosensor, the sidewall Bragg grating waveguide structure based on sensing polymer materials was designed and fabricated based on direct UV writing. The overall schematic diagram of the biosensor is shown in Figure 3. Different concentrations of ginkgolide A ethanol solution can be injected into the microfluid channel and expelled from the tube after flowing across the surface of the waveguide gratings. The ginkgolide A can be selectively adsorbed on the surface of the polymer waveguide grating region effectively. The structural schematic diagram of the proposed waveguide biosensor based on sensing polymer materials is given in Figure 3a.

**Figure 4:**
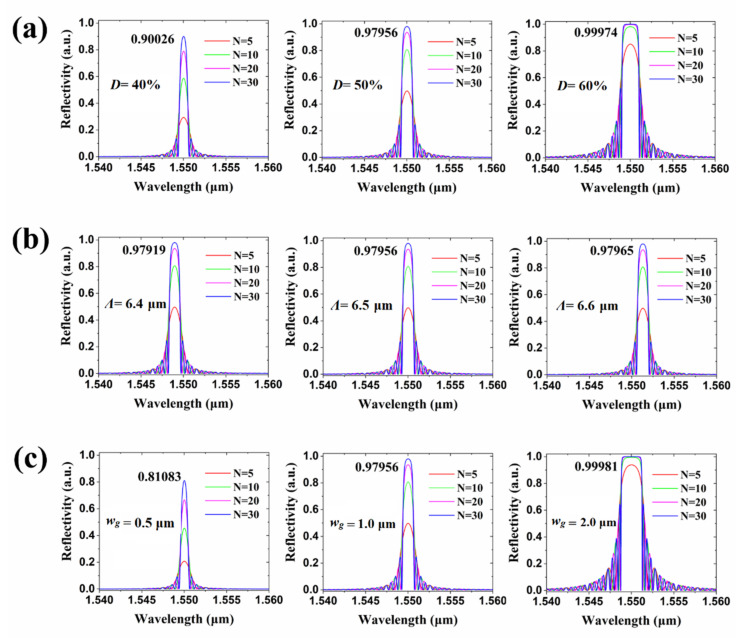
The influences of different waveguide grating duty cycles (*D*) (**a**), periods (*Λ*) (**b**), and corrugation width (*w_g_*) (**c**) of the grating on the reflection spectrum.

Drug solutions in different concentrations are injected into the sensing window by microfluidic channels. As seen in Figure 3b, the period *Λ*, corrugation width (*w*_g_), and duty cycle (*D*) of the Bragg grating are calculated, respectively. The total number of periods for the gratings is analyzed. As shown in Figure 4a–c, the influences of different duty cycles, periods, and corrugation widths of the grating on the reflection spectrum were simulated by MATLAB software, respectively. In the simulation, we set the total number of periods number (N) as 5, 10, 20, and 30, respectively. In Figure 4a, when the grating duty cycles were set as 40%, 50%, and 60%, respectively, the grating reflecting spectra peak amplitude will be enhanced with the increase of duty cycle. It could be observed that high duty cycle will broaden the reflecting spectra of the waveguide gratings, which does not favor the sensitivity for the wavelength shift. Therefore, we chose 50% as the grating duty cycle in sensor design and preparation. Meanwhile, the peak reflectivity of reflecting spectra can reach more than 95%. As shown in Figure 4b, when the period *Λ* of the grating was defined as 6.4 µm, 6.5 µm, and 6.6 µm, respectively, the center wavelength of the reflecting spectra of the grating will produce red-shift with the increase of the grating period. The reference reflecting wavelength of the biosensor we designed is 1.55 µm signal light. Therefore, in terms of different grating period simulation results, we choose 6.5 µm as the designed period, and the peak reflectivity of reflecting spectra can reach more than 95%. In Figure 4c, when the corrugation width of the grating is 0.5 µm, 1.0 µm, and 2.0 µm, respectively, the amplitude of the reflecting spectra peak value of the grating will increase with the increasement of the corrugation width. However, the larger corrugation width will lead to broadening the reflecting spectrum, which is not conducive to the sensitivity of the grating sensor. Therefore, we chose 1.0 µm as the corrugation width of the designed waveguide grating so that the peak reflectivity of reflecting spectra can reach more than 95%. According to the parameters designed for the proposed Bragg gratings, the period *Λ* and duty cycle of the Bragg grating are defined as 6.5 µm and 50%, respectively. The width (*w*) of the waveguide and corrugation width (*w*_g_) of the gratings are set as 2.0 µm and 1.0 µm, respectively. The relationship between the effective refractive index *n*_eff_ of gratings and the Bragg wavelength *λ*_B_ is shown as *mλ*_B_ = 2*Λn*_eff_, where *m* is defined as an order number. When the injected drug solution affects the value of *n*_eff_, the value of *λ*_B_ will also shift. The wavelength change corresponding to the analyte index can be measured to characterize the sensing properties of the sensor.

Based on pharmacological studies, the role of ginkgolide A in cell viability in rat PMVECs and LPS-induced cell cycle in rat PMVECs was analyzed. Rat PMVECs (5 × 10^5^) were incubated with 1–10 µg/mL ginkgolide A for 22 h, followed by treatment with 5 µg/mL lipopolysaccharide (LPS) for 2 h. Total RNA was extracted using the Simply P total RNA extraction kit (Tiangen Biotech Co., Beijing, China), and then cDNA was synthesized using the BioRT cDNA first strand synthesis kit (Bioer Technology Co., Ltd., Hangzhou, China). Sense and antisense primers for c-Myc, Bcl-2, c-Fos, c-Jun, and GAPDH were designed by Premier 5 software. Real-time polymerase chain reaction (RT-PCR) was used to assess the expression levels of the target genes. The primers corresponding to each gene are summarized in Table 1.

“Forward” is referred to as forward prime, and “reverse” is represented as reverse prime. The primes combing to forward sequence gene fragment amplified are defined as forward primes, and those combing to reverse sequence gene fragment amplified are called reverse primes, respectively. RT-PCR is a reaction used for amplifying gene fragments and accessing the expression levels of the target genes, which has one forward prime and one reverse prime, as a pair of primes. In PCR, due to the asymmetry at both ends of DNA molecule, 5′ is phosphoric acid and 3′ is hydroxyl. According to base complementary pairing and semi-reserved replication principle, DNA replication is always from 5′ to 3′. The notation “bp” indicates base pair, and the number of base pairs is used for exhibiting length of DNA or RNA.

**Figure 5:**
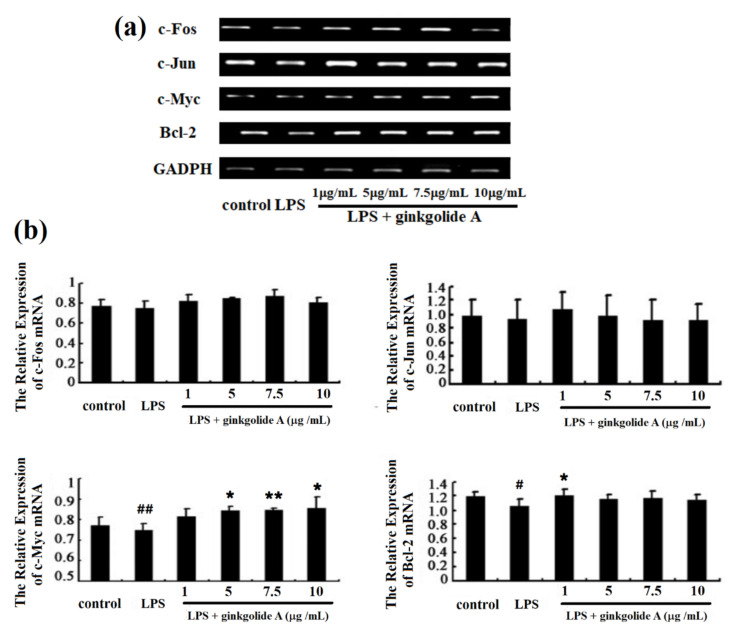
Effects of ginkgolide A on Bcl-2, c-Fos, and c-Jun mRNA expression: (**a**) mRNA levels of c-Myc, Bcl-2, c-Fos, and c-Jun were detected by RT-PCR. (**b**) Values presented are means ± SEM (n = 5 in each group). # *p* < 0.05, ## *p* < 0.01 vs. control group; * *p* < 0.05, ** *p* < 0.01 vs. LPS group.

To assess the effect of ginkgolide A on LPS-induced activation of the NF-κB pathway, c-Myc, c-Fos, c-Jun, and Bcl-2 mRNA expression was measured in rat PMVECs incubated with ginkgolide A in the presence or absence of LPS, as shown in Figure 5a. As seen in Figure 5b, cells treated with LPS had lower mRNA levels of c-Myc, c-Fos, and c-Jun than controls, but the difference was not significant. Compared to cells treated with LPS, cells treated with 5, 7.5, or 10 µg/mL of ginkgolide A showed significantly increased c-Myc mRNA expression. Cells treated with LPS showed significantly lower Bcl-2 mRNA expression than controls. It was found that drug concentrations in the range of 5–10 µg/mL could inhibit PMVEC apoptosis effectively. The parameter *p* in Figure 5b is defined as probability. The parameter *p* < 0.05 indicates that the numerical difference is significant, and *p* < 0.01 shows that the numerical difference is extremely significant, respectively [23].

The actual effective drug concentrations of ginkgolide A ethanol solution for inhibiting PMVEC apoptosis were prepared as targeted analytes. The refractive indices of these drug solutions were measured by an Abbe refractometer and are shown in Table 2.

**Figure 6:**
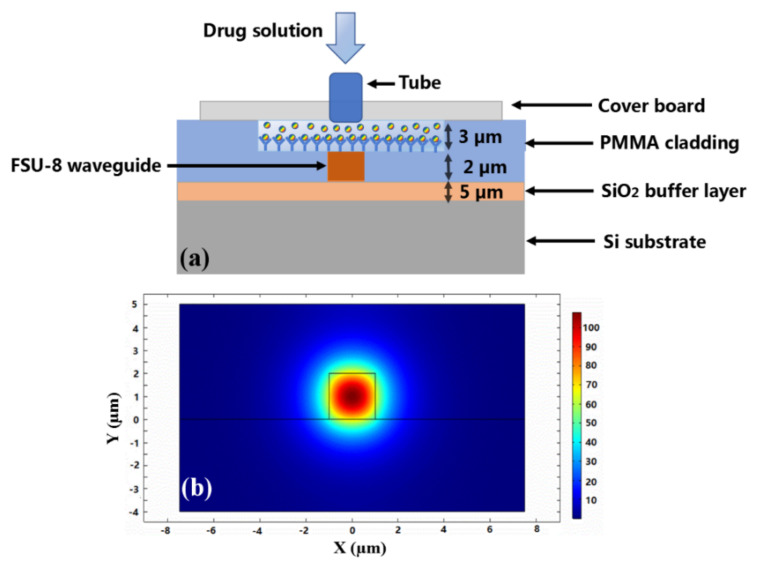
Structural analysis of (**a**) cross−sectional profile of sensing window region of waveguide and (**b**) single−mode optical field distribution calculated based on effective refractive index method.

The cross-sectional structure of the defined waveguide biosensor is shown in Figure 6a. The SiO_2_ layer was grown on Si substrate used as the buffer layer, and the thickness of the oxidized film was 5 µm. The UV photosensitive FSU-8 crosslinked polymer waveguide with 3 µm thickness and 2 µm width was defined on the SiO_2_ buffer layer. The PMMA copolymer with 5 µm thickness was used as the upper cladding. The sensing window with 3 µm depth was formed above the gratings. Through the microfluid channel and into the sensing window, the ginkgolide A solution was absorbed on the surface of the waveguide gratings by hydrogen-bonding affinity. The refractive indices of SiO_2_, FSU-8, and PMMA were 1.4500, 1.5233, and 1.4832 at 1550 nm wavelength, respectively. The effective refractive index *n*_eff_ of the waveguide structure was 1.4903. The fundamental mode distribution of the waveguide was simulated and is shown in Figure 6b. The birefringence of Δ*n*_eff_ between TE and TM mode was about 2 × 10^−4^. The corrugation direction of the designed grating is *x*- direction, so the main polarization of the waveguide mode is the TE polarization mode.

**Figure 7:**
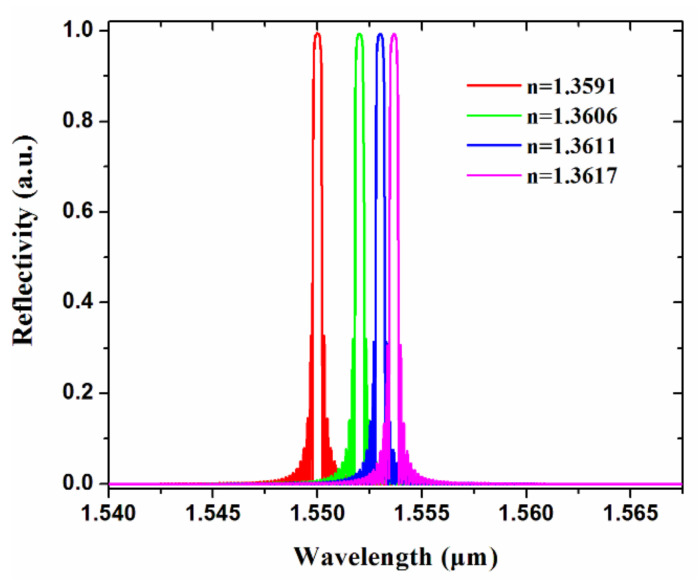
Simulated reflecting spectra of grating-based biosensor corresponding to refractive indices of different concentrations of drug solution.

According to the parameters designed for the proposed Bragg gratings, the reflecting spectra of the chip for different concentrations of drug solution were simulated and are shown in Figure 7. Based on Table 2, when the drug concentration of the solution increases, there is a 4.1 nm red shift for the Bragg reflecting wavelength.

**Figure 8:**
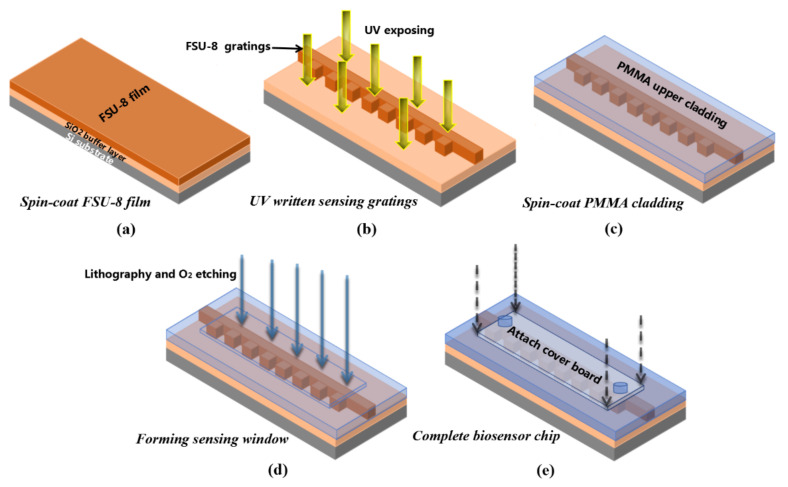
Fabrication process of polymer grating-based biosensor. (**a**) Spin-coat FSU-8 film, (**b**) UV written sensing gratings, (**c**) spin-coat PMMA cladding, (**d**) forming sensing window, and (**e**) complete biosensor chip.

**Figure 9:**
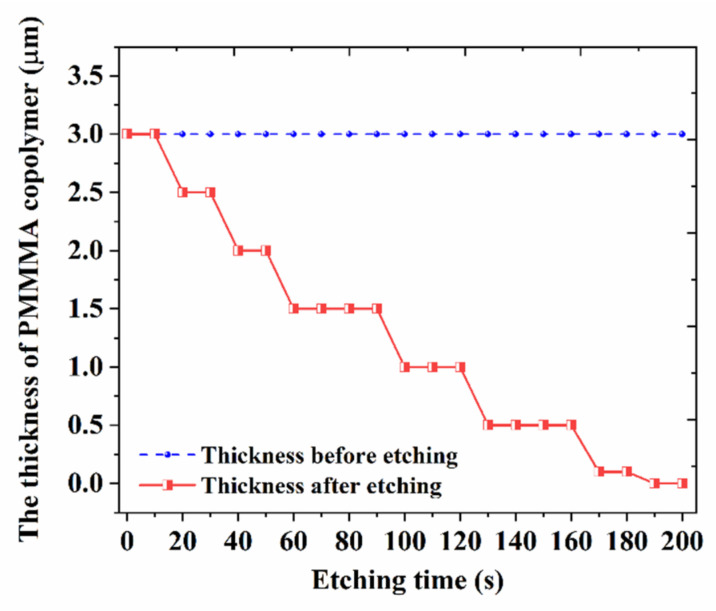
PMMA film thickness after etching corresponding to different film thicknesses and etching times.

The fabrication process of the grating-based biosensor is shown in Figure 8. The FSU-8 negative photopolymer was spin-coated on the SiO_2_ buffer layer of Si substrate. Then, the FSU-8 thin film was cured for 30 min at 95 °C to remove excess solvents and ramped down to room temperature. Next, the FSU-8 waveguide structure was directly written by UV lithography machine (ABM/6/350) at 20 mW/cm^2^ for 6 s, which utilizes contact exposure approach to transfer the photomask patterns into FSU-8 membrane. After that, the sample was post-baked at 100 °C for 1 h, which will excite photoinitiators in FSU-8 to generate enough H^+^, inducing epoxy groups crosslinking. The uncross-linked region of the FSU-8 film was removed with a developer (PGMEA) solution. For the FSU-8 film thickness, the optimized developing time is obtained as 15 s. The average etching rate is about 0.133 µm/s. Then, the PMMA copolymer was spin-coated, covering the waveguide gratings as upper cladding. The PMMA upper cladding layer coating on the surface of the FSU-8 grating was removed through dry etching (reactive ion etching (RIE)). The detailed information of oxygen plasma etching is given as: the flowing rate of O_2_ was 30 sccm, the flowing rate of Ar was 20 sccm, the radio frequency (RF) power of the antenna was 400 W, the bias RF power was 30 W, the pressure was 2.5 Pa, and the pumping vacuum time was 25 s. Figure 9 shows the different PMMA film thicknesses after etching, corresponding to different etching times. The average etching rate is about 0.016 µm/s. The sensing window was well formed on the top surface of the waveguide gratings by controlling the rate of etching. After that, the cover board was bonded onto the sensing window to form the microfluidic channel of the sensor.

**Figure 10:**
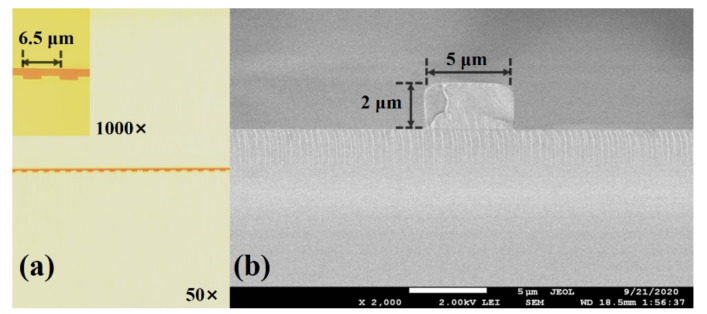
(**a**) Morphology of actual waveguide gratings measured by optical microscope (50× and 1000×) and (**b**) cross-section of input channel waveguide measured by SEM.

The morphology of the actual fabricated waveguide gratings by optical microscope (50× and 1000×) is shown in Figure 10a. It can be found that the designed structural parameters of the chip were achieved. To effectively reduce the mode coupling mismatch between fiber and waveguide, the width of input and output channel waveguide was enlarged to 5 µm using linear-tapering transition. The cross-section of the input channel waveguide was measured by scanning electron microscope (SEM), as shown in Figure 10b. It can be seen that the core size of the waveguide can be well guaranteed.

### 2.3. Biosensor Measurement and Discussion

**Figure 11:**
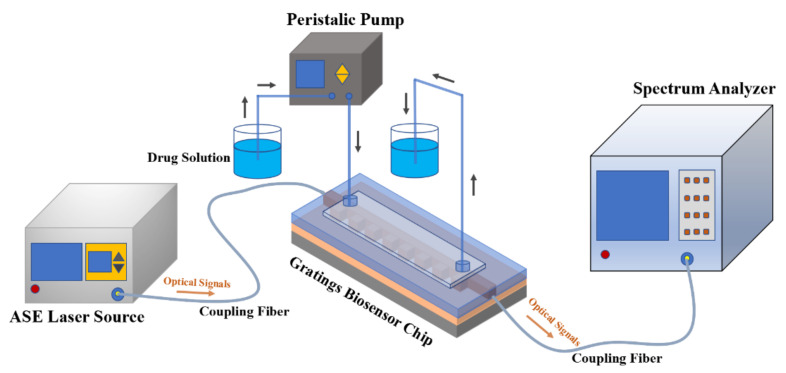
Schematic diagram of measurement system for sensor.

A schematic diagram of the measurement system for the sensor is shown in Figure 11. An amplified spontaneous emission (ASE) light source launches coupled broad-spectrum signal wavelengths from 1510 to 1590 nm by single-mode fiber into the input waveguide of the biosensor. The drug solution is injected into and extracted out of the sensing window region through soft tubes with flow speed controlled by a peristaltic pump. When the testing concentration of ginkgolide A solution is changed, pure ethanol solution is used to wash the sensing window region and tubes continuously for 3 min, which guarantees measuring accuracy every time. The output optical transmission signals were coupled to an optical spectrum analyzer (OSA) by single-mode fiber.

**Figure 12:**
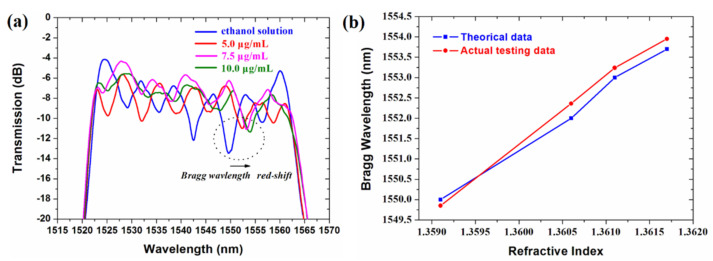
(**a**) Actual measured output spectra of optical signals for grating−based biosensor corresponding to different drug concentrations, and (**b**) comparison of actual and theoretical change curves of Bragg wavelengths related to various refractive indices for different drug concentrations.

The actual output spectra of the optical signals for the biosensor corresponding to different drug concentrations were measured as shown in Figure 12a. It was found that the reflecting Bragg wavelength underwent a red-shift phenomenon with increased drug concentration of ginkgolide A solution. The total insertion loss of the biosensor was obtained as 4.1 dB. The contrast of the grating device was about 10 dB. The actual and theoretical change curves of Bragg wavelengths related to various refractive indices for different drug concentrations are given in Figure 12b. Based on the linear fitting method, it can be calculated that the theoretical sensitivity of the sensor was 1443.7 nm/RIU, and the actual sensitivity was 1606.2 nm/RIU. Regarding sensitivity, the actual measured value was larger than that of the theoretical one. The reason may be that, compared to the results of capturing drug monomer from the molecular docking simulation, there was some extra binding force between ginkgolide A and the polymer waveguide materials, including FSU-8 and PMMA, and generated by the RIE oxygen ion bombardment process. The relationship between the sensitivity *S* and the limit of detection (LOD) *L* for the biosensor is given as *L* = *R*/*S*, where the sensor resolution *R* is related to system noise. The resolution of the biosensor can be estimated by *R* = 3*σ*, where σ is the standard deviation of the measured wavelength shift for the sensor as the output noise of system. The resolution *R* was obtained as 0.05 nm, and detection limit *L* was about 3 × 10^−5^ RIU.

Compared with other reported grating-based biosensors, the operating area and sensitivity values in this work were contrasted with those published in the literature (Table 3). It can be observed that there is a relatively small operating area and large sensitivity for the proposed biosensor. The merits of the overall parameters for the proposed biosensor can be clearly seen.

## 3. Conclusions

In summary, a fluorinated copolymer Bragg waveguide grating-based optical biosensor to detect effective solution concentrations of ginkgolide A for inhibition of PMVEC apoptosis was achieved by the direct UV writing technique. Low-loss FSU-8 and PMMA were used as the sensing polymer waveguide materials. The stable binding energy based on hydrogen-bonding affinity enhanced the capability of adhesion and capture between ginkgolide A and the polymer waveguide materials. The effective drug concentration range (5–10 µg/mL) of ginkgolide A for inhibiting PMVEC apoptosis was analyzed and obtained by pharmacological studies. The actual sensitivity of the grating-based biosensor was measured as 1606.2 nm. The resolution was obtained as 0.05 nm, and the detection limit was about 3 × 10^−5^ RIU. The proposed technique is suitable for detecting organic drug monomers such as ginkgolide A. This type of polymer waveguide grating-based biosensor is advantageous to realize lab-on-chip integrated photonic circuits and could potentially be used for secure and accurate detection of Chinese herbal ingredients.

## Figures and Tables

**Table 1:** Primer sequences used in real-time polymerase chain reaction.

Name	Sequence	Length
c-Fos	forward 5′-CCCGTAGACCTAGGGAGGAC- 3′reverse 5′-CAATACACTCCATGCGGTTG- 3′	189 bp
c-Jun	forward 5′-ACGCCAACCTCAGCAACTT- 3′	192 bp
	reverse 5′-TCTGCGGCTCTTCCTTCA- 3′	
c-Myc	forward 5′-ACAACCGCAAATGCTCCA- 3′	259 bp
	reverse 5′-CGCCGTTTCCTCAGTAAGTC- 3′	
Bcl-2	forward 5′-GGCATCTTCTCCTTCCAGC- 3′	476 bp
	reverse 5′-TCCCAGCCTCCGTTATCC- 3′	
GAPDH	forward 5′-CACTGCCACTCAGAAGACT- 3′	177 bp
	reverse 5′-ACATTGGGGGTAGGAACAC- 3′	

**Table 2:** Refractive indices of ginkgolide A ethanol solution with different concentrations.

	Ginkgolide A Ethanol Solution			
Ginkgolide A concentration (µg/mL)	0	5.0	7.5	10.0
Refractive index of drug solution	1.3591	1.3606	1.3611	1.3617

**Table 3:** Comparison with results published for other waveguide grating-based biosensors.

Type of Device	Analyte	Operating Area (μm^2^)	Sensitivity	Reference
Racetrack grating	Glycerol	314	429 nm/RIU	[24]
Microdisk grating	NaCl	114	390.4 nm/RIU	[25]
Microring grating	Glucose	80	363 nm/RIU	[26]
Linear grating	Ginkgolide A	260	1606.2 nm/RIU	This work

## Data Availability

Not applicable.
